# Combined application of dinitrofluorobenzene and ovalbumin induced AD-like dermatitis with an increase in helper T-cell cytokines and a prolonged Th2 response

**DOI:** 10.1186/s12865-022-00531-2

**Published:** 2022-12-07

**Authors:** Pengju Jiang, Yaguang Wu, Lu Liu, Lian Zhang, Zhiqiang Song

**Affiliations:** 1 School of Medicine, Chongqing University, Chongqing, 400030 China; 2 Department of Dermatology, Southwest Hospital, Army Medical University, Chongqing, 400038 China

**Keywords:** Atopic dermatitis, Dinitrofluorobenzene, Ovalbumin, BALB/c mice

## Abstract

**Background:**

The progression of acute-to-chronic atopic dermatitis is accompanied by multiple helper T-cell cytokine responses, but the mechanisms and relative importance of these changes remain unclear. There is no animal model for atopic dermatitis that recapitulates these cytokine responses.

**Objective:**

We sought to build a novel mouse model for atopic dermatitis (AD) that recapitulates these helper T-cell responses and some dynamic changes in cytokine responses in the progression of AD.

**Methods:**

Female BALB/c mice were subjected to the application of dinitrofluorobenzene (DNFB) and ovalbumin (OVA) to induce AD-like dermatitis. Skin lesions and serum were collected from mice in the acute and chronic phases to detect changes in cytokine responses and other features of AD.

**Results:**

Combined application of DNFB and OVA successfully induced AD-like dermatitis and histological changes as well as epidermal barrier dysfunction. In the acute phase of AD-like dermatitis, Th2-associated cytokines were mainly increased in serum and skin lesions. In the chronic phase of AD-like dermatitis, Th2-associated cytokines were still highly expressed, while Th1- and Th17-associated cytokines were also gradually increased. Compared with the acute phase, the JAK-STAT signaling pathway was highly expressed in the chronic phase of AD-like dermatitis.

**Conclusion:**

The combined application of DNFB and OVA could be used to build a new mouse model for atopic dermatitis. This mouse model recapitulates the helper T-cell responses and some dynamic changes in cytokine responses in the progression of acute-to-chronic in human AD. The JAK-STAT signaling pathway plays a pivotal role in the chronicity of AD.

## Background

Atopic dermatitis (AD) is one of the most common skin diseases that predominantly manifests as chronic inflammation, affecting up to 10-20% of children and adults in developed countries [1, 2], and the prevalence is still increasing. Many patients with AD also suffer from allergic rhinitis and asthma along with intense itching and skin infection [3]. The hallmark clinical sign of AD is eczematous lesions, which may present acutely or chronically. It is unclear whether the chronic and acute phases of AD are driven by different immune pathways or just represent a different extent of inflammatory response. AD is a result of skin barrier disruption and an imbalance in the innate and adaptive immune systems [4]. Data from human and animal studies suggest that AD is a Th2-dominant inflammatory skin disease at the acute stage followed by Th1 and Th17 involvement at the chronic stage[5, 6]. Activated Th2 cells release IL-4 and IL-13, inducing IgE class switching in B cells and the secretion of antigen-specific IgE through the JAK-STAT signaling pathway, which may lead to the chronicity of AD[7].

Over the last several decades, many kinds of animal disease models have enabled us to deepen our understanding of the pathogenesis of AD[8]. Epidermal barrier dysfunction, skin-laden immune cells, and keratinocytes all play critical roles in the progression of AD. Thus, AD animal models are frequently generated by the intraperitoneal application of exogenous allergens[9, 10]. Human and animal skin exhibit many species-specific differences, such as the tissue architecture, frequency of hair cycling, immune responses and percutaneous penetration[11]. To date, there is no animal model for AD that comprehensively recapitulates all aspects of human AD characteristics. Most existing mouse models for AD, such as the MC903 mouse model and the Dermatophagoides farinae mouse model, only induce AD-like dermatitis and Th2-associated cytokine responses[12]. An ideal animal model for AD would recapitulate as many features of human AD as possible, including keratinocyte hyperproliferation, thickening and altered differentiation of the epidermis, increased T-cell infiltration, elevated Th2/Th1 cytokine expression and serum IgE levels and disrupted skin barrier[13]. Given that little is known about the transition of AD from the acute to chronic stages, further investigation of the mechanisms behind this transition requires a new mouse model.

Exposure to dinitrofluorobenzene (DNFB) was found to induce contact dermatitis in mice[14]. When the shaved back skin of mice is exposed to DNFB, cytotoxic T lymphocytes are recruited to the skin and result in keratinocyte apoptosis and further skin barrier dysfunction[15]. Ovalbumin (OVA) is a chicken protein allergen mainly found in egg white that is commonly used to sensitize immune reactions[9]. Prolonged exposure to OVA induces the development of an AD-like dermatitis characterized by OVA-specific IgE and total serum IgE, as well as increased expression of Th2 cytokines and IFN-γ. However, the induction period of AD-like dermatitis using OVA is time-consuming, and the skin lesions are usually mild. Therefore, we designed this study to evaluate the potential of the combined application of DNFB and OVA to build a new AD mouse model that recapitulates the dynamic changes in helper T-cell responses in the progression of acute-to-chronic atopic dermatitis. In this study, female BALB/c mice were subjected to the application of DNFB three times a week and OVA twice a week, and the same protocol was repeated for 4 weeks. The mice in the experimental group developed an impaired skin barrier and typical cutaneous manifestations of AD, and they exhibited classic pathological features, as evidenced by the prominent infiltration of inflammatory cells and epidermal thickening. The expression of Th2-associated cytokines (IL-4, IL-13, and TSLP) was elevated in the acute stage, while in the chronic stage, the expression of Th2-associated cytokines was still at a high level, while the expression of Th1- and Th17-associated cytokines was also prominently elevated. In addition, high JAK-STAT signaling pathway expression was also observed in the chronic stage of this mouse model.

## Results

### Combined application of DNFB and OVA induced AD-like dermatitis

**Fig. 1:**
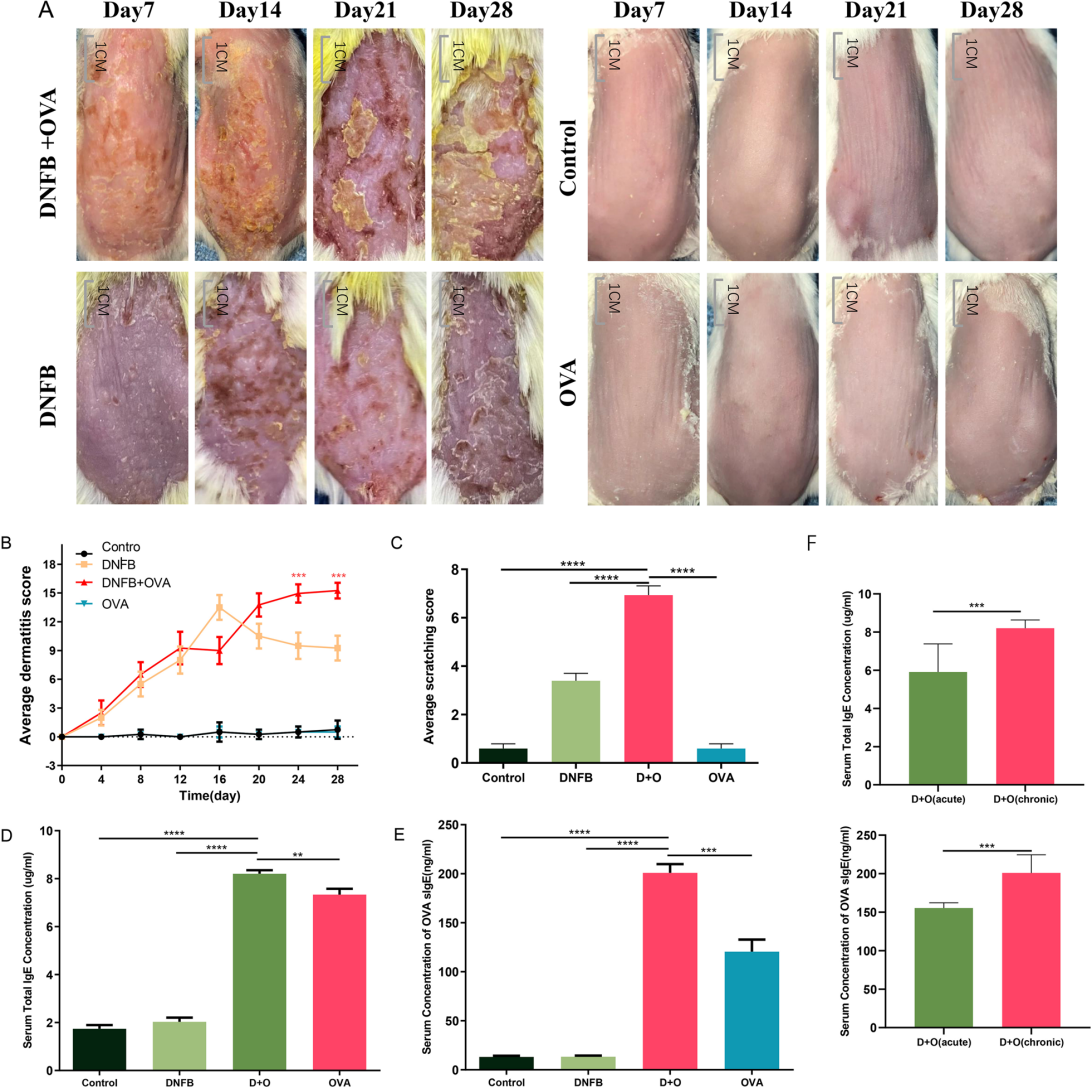
Typical AD-like dermatitis and scratching behavior were induced by DNFB + OVA. **A** Morphological changes of skin lesion during four weeks. **B** Skin lesion scorings. The total scores of day 24 and day 28 were compared between D + O group and DNFB group. **C** Scratching behavior examination. **D**, **E** The serum total IgE and OVA-specific IgE in each group. **F** Serum IgE concentration of acute phase and chronic phase in the DNFB + OVA group. (Date shows mean ± SD, significance levels of data were denoted as *P < 0.05, **P < 0.01, and ***P < 0.001, ****P<0.0001)

We successfully induced AD-like dermatitis by alternatively using DNFB and OVA. As vividly presented in Fig. 1 A, we can see the dynamic changes in skin lesions in each group. With prolonged induction time, the skin lesions of mice in the DNFB group tended to be stabilized, which predominantly presented with dryness, erythema and scaling. However, the skin lesions of mice in the DNFB + OVA group were consistently exacerbated with increasing exposure time, as evidenced by skin thickening, erythema area enlargement and severe edema and exudation. To compare the morphological changes in skin lesions between different groups, skin lesion scoring was performed and recorded every four days (Fig. 1B). After approximately two weeks, mice in the DNFB group exhibited dryness, scaling, and poorly defined erythema, but neither edema nor exudation was observed. However, in the DNFB + OVA group, apart from erythema, scaling and dryness, mild edema and exudation were also observed. At the end point of this experiment, scratching behavior estimation was performed, and the results indicated that the scratching scores of mice in the DNFB + OVA group were prominently higher than those in the other groups (Fig. 1 C). As illustrated in Fig. 1D, the serum total IgE levels of the DNFB + OVA group were 3.89-fold (P<0.0001) and 1.17-fold (P<0.01) higher than those of the DNFB group and OVA group, respectively. As shown in Fig. 1E, the serum OVA-specific IgE levels of the DNFB + OVA group were markedly higher than those of the DNFB and OVA groups. Figure 1 F shows the comparison of serum IgE levels between the acute phase and chronic phase within the DNFB + OVA group. Overall, compared to those in the other groups, the skin lesions of mice in the DNFB + OVA group were the most severe, whereas no significant skin lesions were observed in the OVA and control groups during the entire induction period.

### Combined application of DNFB and OVA induced AD-like histological changes

**Fig. 2:**
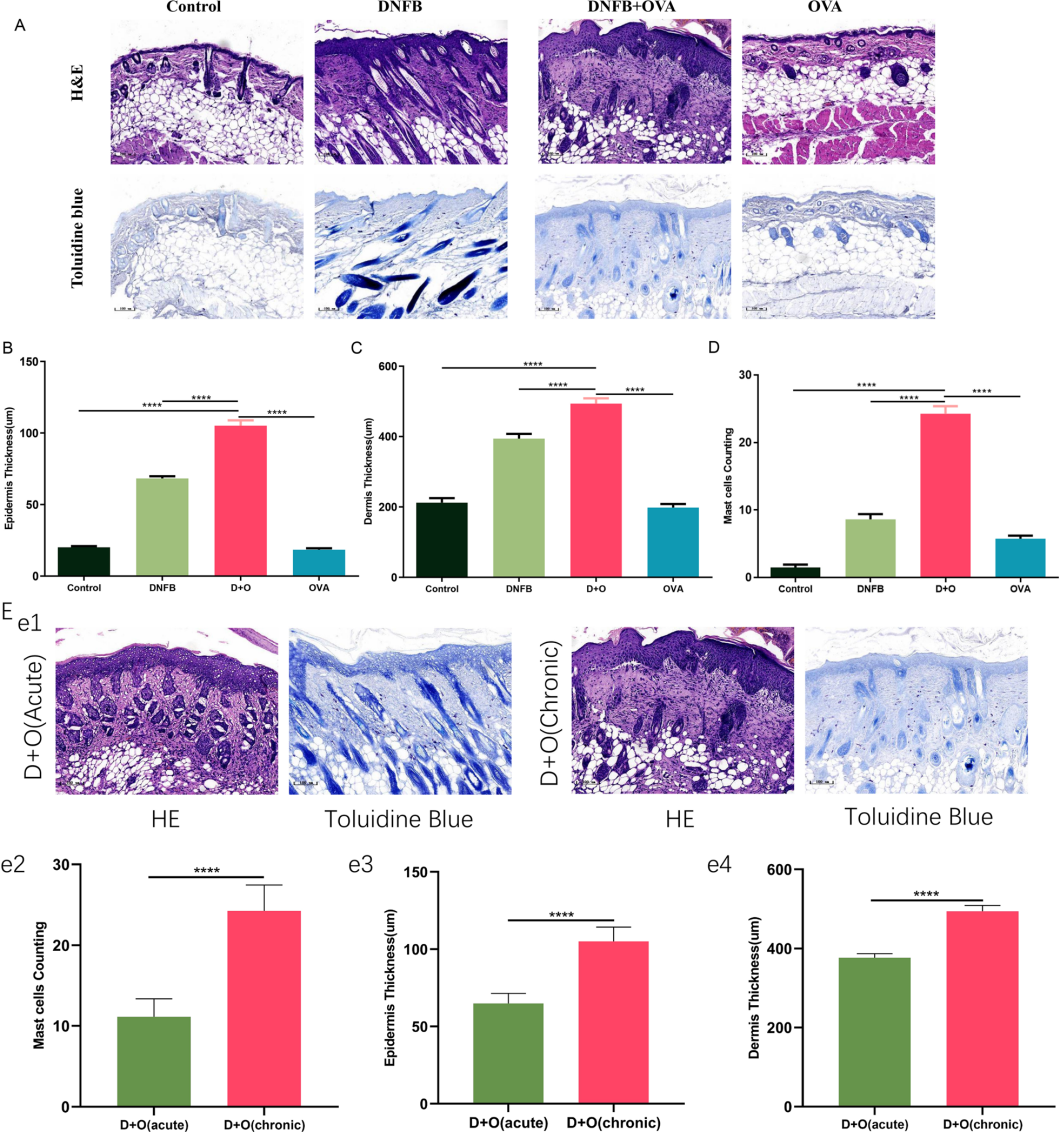
DNFB + OVA induced typical AD-like phenotypic and histologic changes in BALB/C mice. **A** Representative images of H&E and Toluidine blue stained sections from each group; scale bar = 100 μm. **B**, **C** Comparison of the epidermal and full thickness of skin in mice subjected to different treatments. **D** The average numbers of mast cells per field. Data are representative of five randomly selected fields from each section. **E** Comparison of histological changes between acute phase and chronic phase in the DNFB + OVA group. (Date shows mean ± SD, significance levels of data were denoted as *P < 0.05, **P < 0.01, and ***P < 0.001, ****P<0.0001)

To probe the histological changes among these groups, sections were stained with H&E and toluidine blue to examine the epidermal thickness and the infiltration of mast cells, respectively. In acute lesions of the DNFB + OVA group, spongiosis and edema were slightly more severe than in those of other groups; however, when the dermatitis became chronic, lesions in the DNFB + OVA group showed more prominent lichenification, thickening of the epidermis and less pronounced spongiosis than in other groups (Fig. 2 A–C). Mast cell infiltration was analyzed in toluidine blue-stained sections using Iviewer software, and mast cell counting was performed in five randomly selected fields from each section. As illustrated in Fig. 2D, the infiltration of mast cells in the DNFB + OVA group was markedly higher than that in any other group. Figure 2E shows the comparison of histological changes between the acute phase and chronic phase within the DNFB + OVA group. Clearly, the inflammatory cell infiltration and skin thickening in chronic lesions were both more severe than those in the acute phase.

### Combined application of DNFB and OVA aggravates epidermal barrier dysfunction

**Fig. 3:**
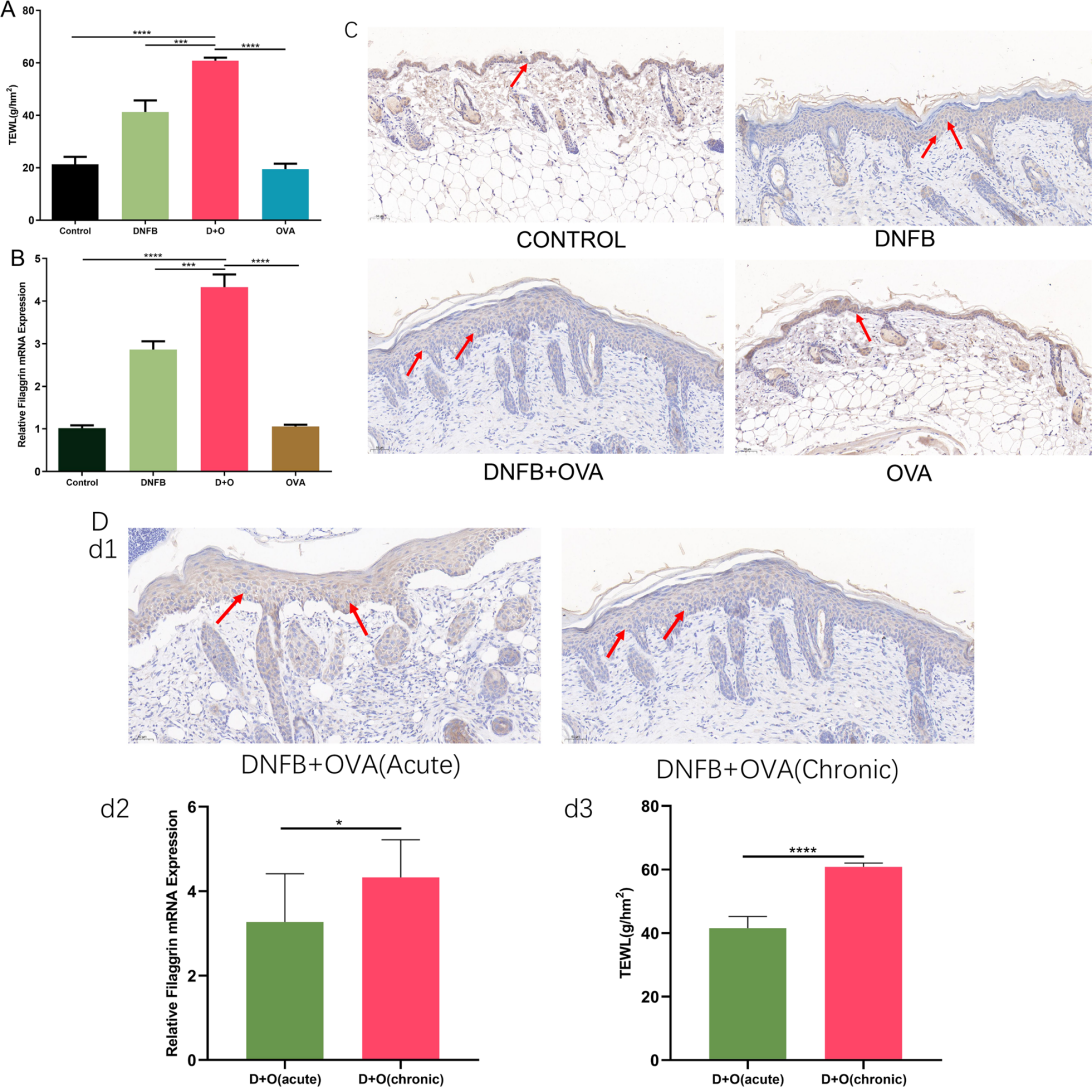
DNFB + OVA induced epidermal barrier dysfunction and higher FLG mRNA expression. **A** TEWL values in each group. **B** The comparison of the expression levels of FLG gene between each group. **C** Representative sections of anti-filaggrin immunohistochemistry staining in each group. Typical positively stained cells are marked with arrows. **D** (d1) The comparison of positively stained area between acute lesions and chronic lesions. (d2,d3) The comparison of FLG mRNA expression levels and TEWL values between acute lesions and chronic lesions. (Data shows mean ± SD. Scale bar = 50 μm. significance levels of data were denoted as *P < 0.05, **P < 0.01, and ***P < 0.001, ****P<0.0001)

As Fig. 3A illustrates, our data suggest that the TEWL values of the DNFB + OVA group were 1.48-fold and 2.97-fold higher than those of the DNFB group and OVA group, respectively (*P*<0.001). In addition, our data confirm that the application of DNFB + OVA upregulated filaggrin expression in the skin tissue of mice (Fig. 3B). To explore the mechanism behind this finding, we performed immunohistochemistry to investigate the protein expression level of filaggrin, and the results (Fig. 3C) showed that the number of positively stained cells in the DNFB + OVA group was not higher than that in the DNFB group but was much higher than that in the control and OVA groups. In contrast to the high mRNA expression level, the number of positively stained cells in the epidermal layer of the D + O group was not higher than that in the other groups, which is an intriguing but puzzling phenomenon. Given the highest TEWL values in chronic lesions of the D + O group, its most severely damaged skin barrier may be responsible for this phenomenon, but the precise mechanism deserves further investigation. Figure 3D shows the comparison of epidermal barrier-related indicators between the acute phase and chronic phase within the DNFB + OVA group. d1: The filaggrin-positive staining area in the epidermis of chronic skin lesions was less than that of the acute phase. d2: The mRNA expression in chronic lesions was higher than that in acute lesions. d3: The TEWL values in chronic lesions were much higher than those in acute lesions.

### AD-like dermatitis is dominated by the Th2 immune response

**Fig. 4:**
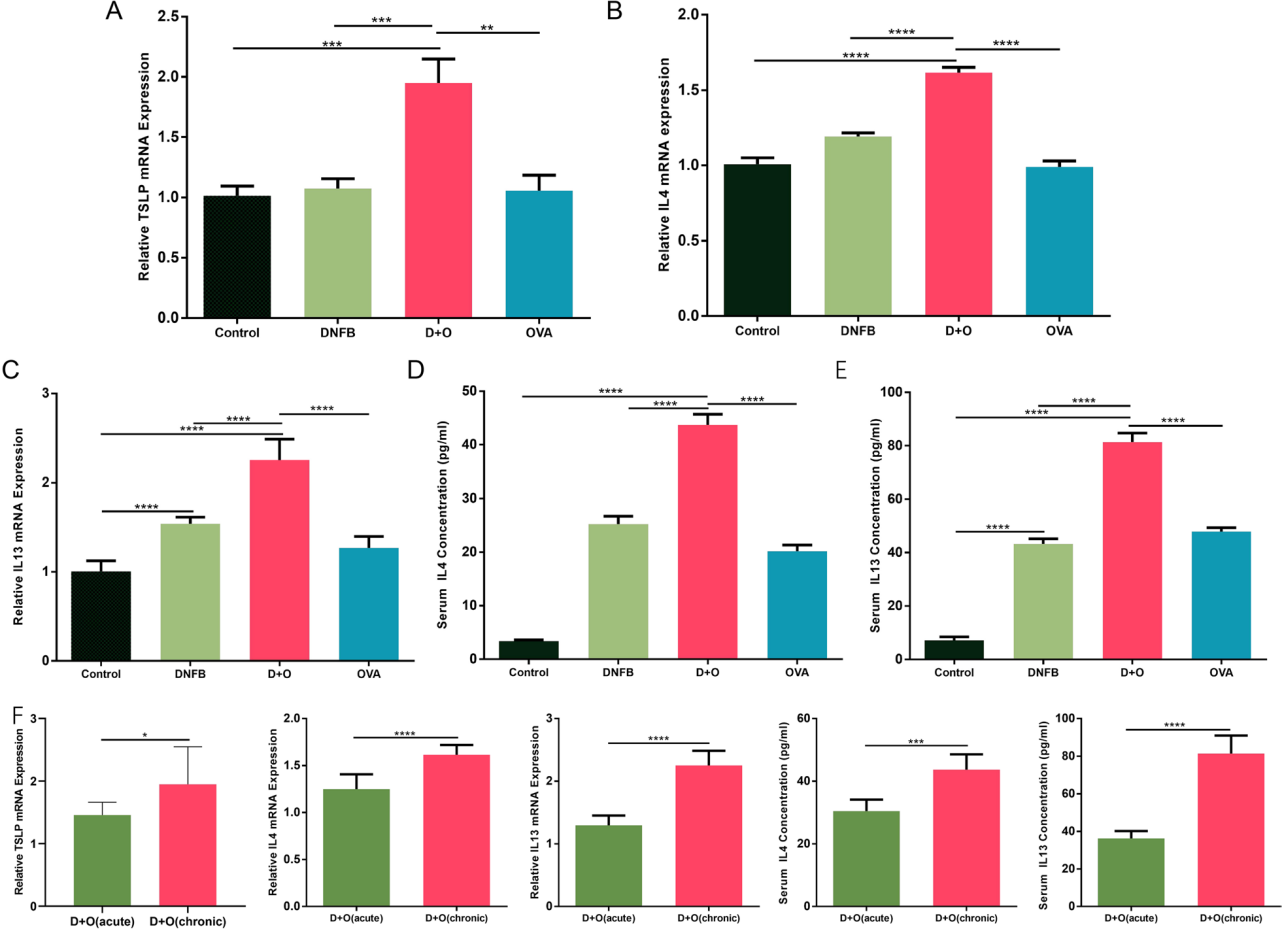
Combined application of DNFB + OVA aggravates the expression levels of Th2 and Th2 related cytokines both in serum and skin lesions. **A**–**C** The gene expression levels of TSLP, IL4, IL13, respectively. **D**, **E** Serum concentrations of IL4 and IL13 in each group. **F** Comparison of Th2 related cytokines between acute phase and chronic phase in the DNFB + OVA group. (Data shows mean ± SD. significance levels of data were denoted as *P < 0.05, **P < 0.01, and ***P < 0.001, ****P<0.0001)

Atopic dermatitis is usually considered a T helper 2 (Th2) cell-mediated disease. In addition, as a master regulator of Th2-driven inflammation, TSLP promotes the early differentiation of distinct Th2 cell populations. Activated Th2 cells release IL-4 and IL-13, which together aggravate the inflammatory cascade. As shown in Fig. 4A, our data showed that the expression level of TSLP in the DNFB + OVA group was 1.92-fold higher than that in the control group (P<0.001). In addition, DNFB + OVA also enhanced the expression of IL-4 and IL-13 both in serum and skin lesion tissues (Fig. 4B–E), albeit the enhancement of IL-4 was more subtle. As shown in Fig. 4F, within the DNFB + OVA group, the expression of Th2-related cytokines in the chronic phase was also higher than that in the acute phase, which further confirms that the Th2 immune response dominates the progression of atopic dermatitis.

### The expression of helper T-cell-associated cytokines is upregulated in the chronic stage of AD-like dermatitis

**Fig. 5:**
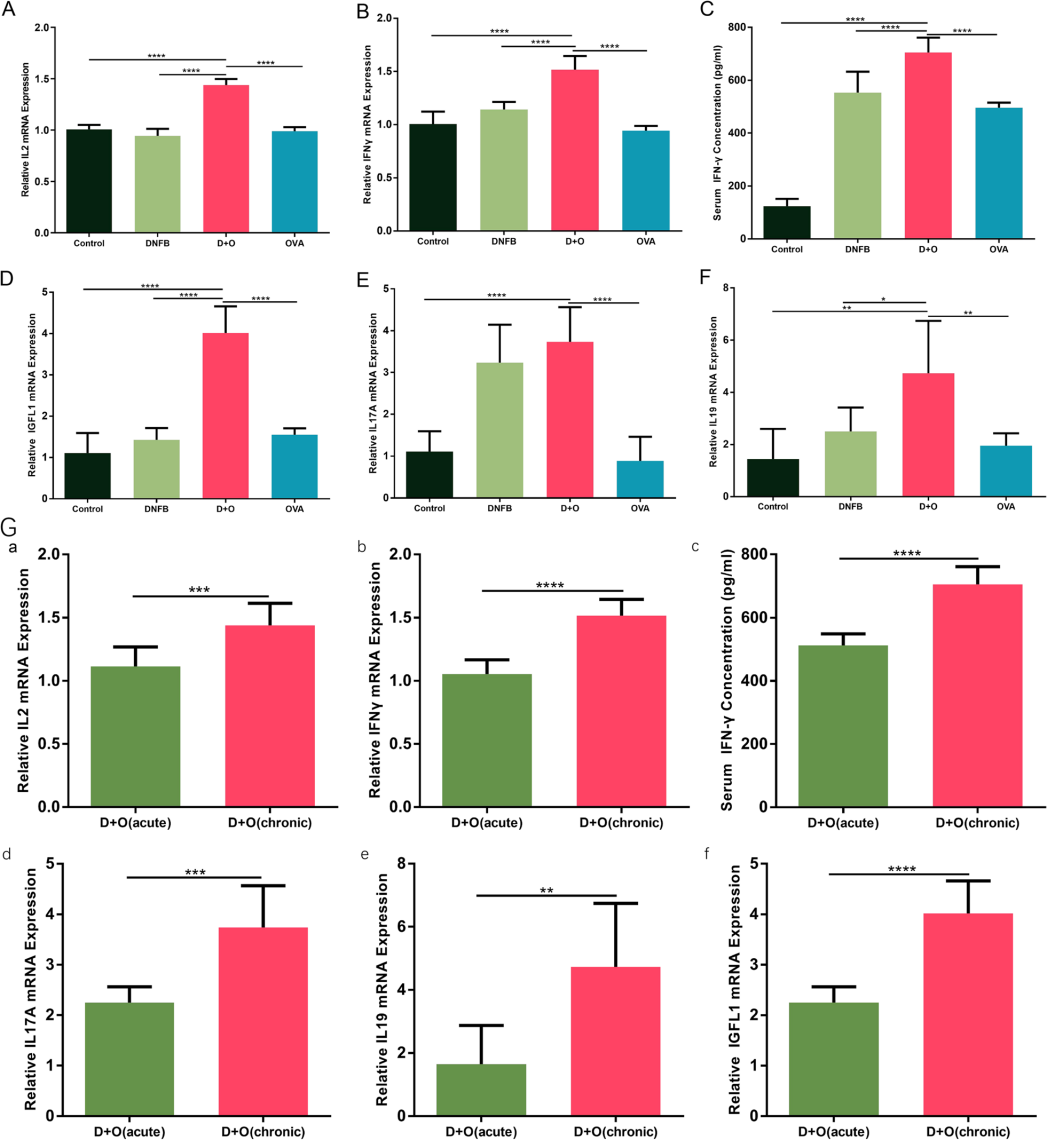
Upregulation of Th1 and Th17 related cytokines were observed in DNFB + OVA group. **A**, **B** The gene expression levels of Th1 related cytokines. **C** Serum concentration of IFN-γ. **D**, **E** Gene expression levels of IL17A and IGFL1 in each group. **F** Gene expression level of IL19 in each group. **G** Comparison of Th1/Th17 related cytokines expression between acute phase and chronic phase in the DNFB + OVA group. (Data shows mean ± SD. significance levels of data were denoted as *P < 0.05, **P < 0.01, and ***P < 0.001, ****P<0.0001)

Recently, upregulation of Th1- and Th17-mediated responses has been found to be present in chronic skin lesions[16]. Given that chronic AD is strongly associated with the enhanced expression of Th1- and Th17-related cytokines, we also examined these cytokines in skin tissues and serum using PCR and ELISA. Figure 5A–F illustrates the comparison of helper T-cell-associated cytokine expression between different groups at the end of the experiment. As illustrated in Fig. 5G, we compared helper T-cell-associated cytokine expression in the acute and chronic phases within the DNFB + OVA group. To be specific, panels a and b in Fig. 5G show that the expression levels of Th1-related cytokines were higher (1.22-fold, *P*<0.001; 1.38-fold, *P*<0.001; and 1.31-fold, *P*<0.001, respectively) in chronic lesions than in acute lesions within the DNFB + OVA group; and panel d in Fig. 5G shows the comparison of the expression levels of IL-17 A, which were also higher in chronic lesions of the DNFB + OVA group. As we can tell from panel e and f in Fig. 5G, the expression levels of IL-19 and IGFL1 (a newly found cytokine that is closely related to the activation of IL-17) were also higher (2.67-fold, *P*<0.01; and 1.73-fold, *P*<0.001, respectively) in the chronic lesions of the DNFB + OVA group.

### Upregulation of the JAK-STAT pathway in chronic lesions

**Fig. 6:**
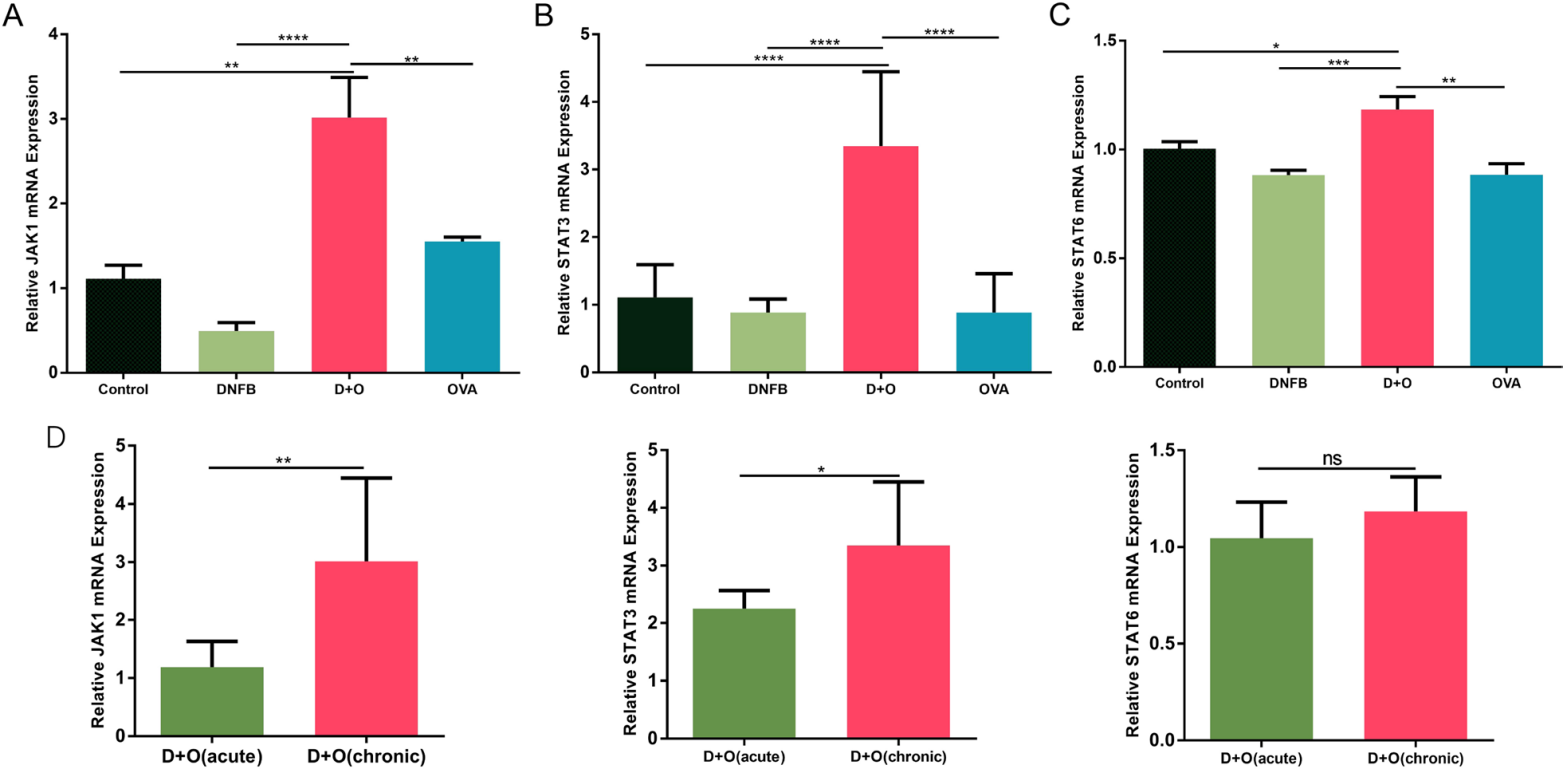
Comparisons of the JAK-STAT signaling pathway expression levels in mice subjected to different treatments. **A**–**C** The expression levels of JAK-STAT pathway in each group. **D** Comparison of the expression levels of JAK-STAT pathway between acute phase and chronic phase in the DNFB + OVA group. (Data shows mean ± SD. Significance levels of data were denoted as *P < 0.05, **P < 0.01, and ***P < 0.001, ****P<0.0001)

It is well known that activated Th2 cells secrete IL-4 and IL-13 and that these cytokines promote IgE class switching in B cells and the production of IgE via the JAK-STAT signaling pathway, which may lead to the chronicity of AD. Thus, we quantified the expression levels of JAK-STAT signaling pathway intermediates. As Fig. 6A–C shows, our data indicate that the application of DNFB + OVA increased the expression levels of JAK1 (2.89-fold, *P*<0.001), STAT3 (3.47-fold, *P*<0.001), and STAT6 (1.23-fold, *P*<0.05) compared to the levels in the control group. As presented in Fig. 6D, our data indicate that the expression levels of JAK1 and STAT3 in chronic lesions of the DNFB + OVA group were also higher than those in acute lesions.

## Discussion

Atopic dermatitis is one of the most common skin diseases and poses a significant socioeconomic and public health burden [17]. AD affects people of all ages and ethnicities and has a severe physical and mental impact on patients and families. The pathogenesis is highly complicated and involves strong T-cell-driven inflammation, epidermal barrier dysfunction, and genetic predisposition [18]. Currently, in the field of AD research, mouse models are the most commonly used model system due to a multitude of favorable properties, such as ease of raising, rapid breeding and inbred capability. However, because mice and humans exhibit many species-specific differences in immune responses, skin tissue architecture and percutaneous drug penetration [11], there is no mouse model that recapitulates the dynamic changes in the cytokine response in the progression of AD.

In this study, female BALB/c mice were subjected to application of DNFB and OVA for 4 consecutive weeks. The mice in the DNFB + OVA group developed an impaired skin barrier and typical cutaneous manifestations of AD, including erythema, edema, scaling, and a high frequency of scratching, and they also exhibited classic pathological features, as evidenced by the prominent infiltration of inflammatory cells and epidermal thickening. At the second week, mice in the DNFB group exhibited dryness, scaling, and poorly defined erythema, but neither edema nor exudation was observed. However, in the DNFB + OVA group, apart from erythema, scaling and dryness, mild edema and exudation were also observed. With prolonged induction time, the skin lesions of mice in the DNFB + OVA group tended to be stabilized and chronic, which predominantly presented with dryness, erythema and scaling. In terms of the cytokine response, at the acute stage, the immune response was dominated by Th2-promoting and Th2 cytokines, namely, TSLP, IL-13 and IL-4, while after AD-like dermatitis progressed to its chronicity, other cytokine responses, such as Th1- or Th17-related cytokines, IGFL1 and IL-19, were also markedly elevated.

The immune responses of atopic dermatitis are a complex interplay between CD4 + T-cell activation and differentiation, the Th1/Th17 immune pathway, and mainly type 2 skewed immune dysregulation [19]. The type-2 immune response is dominant in the acute phase of AD, mainly manifested as increased levels of IL-4 and IL-13, as well as increased serum IgE concentrations [20]. As a Th2-related cytokine, TSLP is greatly involved in the pathogenesis of AD. First, after its release in keratinocytes, TSLP activates tissue-resident DCs that subsequently migrate into the local lymph node, where they prime naïve CD4 + T cells into Th2 cells [21]. TSLP further triggers the secretion of proinflammatory cytokines such as IL-4, IL-13 and TNFα from CD4 + T cells and DCs, which further activate B cells and mast cells [22]. It has been reported that at the chronic phase of atopic dermatitis, activation of Th1- and Th17-mediated responses has been observed [16], but the relative importance of these pathways in atopic dermatitis is currently unclear. Among all the mouse models of atopic dermatitis, our model first recapitulates some dynamic changes in Th-related cytokines in the progression of AD from the acute phase to the chronic stage.

Our results also showed that JAK-STAT pathway expression was significantly upregulated in chronic lesions. Janus kinases (JAKs) are a family of cytoplasmic tyrosine kinases (TYKs) comprising JAK1, JAK2, JAK3, and TYK2. The JAK-STAT pathway plays a central role in the maturation of B-lymphocyte cells [23]. In vitro studies indicate that inhibitors targeting JAK1 decrease the number, activation, and function of T cells, B cells, DCs, and mast cells [24]. Given that these cells are greatly involved in the progression of AD, among all JAKs, JAK1 plays a more important role in the pathogenesis of AD. The STAT family comprises STAT1, STAT2, STAT3, STAT5A/B, and STAT6. STAT6 is involved in B-cell differentiation, IgE class switching, and MHC class II production; therefore, STAT6 is associated with IgE production and the progression of allergic diseases [25]. STAT3 is vital for Th17 lymphocyte differentiation, and STAT3 is also involved in mediating the inflammatory process and changes in natural skin barriers, as well as increasing TEWL by stimulating the expression of IFN-γ, IL-31, and IL-22 [26, 27]. Our results confirm that the expression of the JAK-STAT pathway was markedly increased in both the acute and chronic phases, and the increase was more significant in the chronic phase. Taken together, we speculate that the high expression of the JAK-STAT pathway may promote the chronicity of AD.

Increased IL-19 expression is usually observed in inflammatory skin, such as atopic dermatitis and aging [28]. The Th17-related cytokines IL-17 A and IL-22 act synergistically to enhance IL-19 expression in cultured keratinocytes in vitro [28]. Since Th17-related cytokines are a marker of chronic AD, IL-19 may also be an indicator of the chronicity of AD. IGFL1 has been reported as a candidate target gene for AD, and it may be related to the abnormal activation of the immune response in AD patients, especially the activation of Th17 immunity, which is one of the key subtypes of the T-cell pathway [29]. Since IL-19 and IGF1 were associated with Th17 cells and our results also showed that the expression of both IL-19 and IGFL1 was upregulated in chronic lesions, we speculated that IL-19 and IGFL1 might be new indicators of AD chronicity.

## Conclusion

Our findings suggest that the combined application of DNFB + OVA successfully induced AD-like skin lesions and histological findings in BALB/c mice with a rather short induction period. This model also partially recapitulates the dynamic changes in the cytokine response in the acute-to-chronic progression of AD.

## Limitations

This study has potential limitations. Although this study recapitulates some of the dynamic changes accompanying the transition from acute to chronic inflammation in AD, according to previous transcriptomic experiments, it is far from comprehensively representing human AD signatures. Using patient-derived samples, Tsoi et al. reported that the transition to chronic AD is associated with elevated Th2, Th1, Th17, and IL-36 responses, and they also found that FOXK1 (a transcription factor that modulates developmental and cell differentiation processes) may promote the chronicity of AD[30]. In addition, Gittler et al. reported that S100 family members are closely related to acute AD, particularly S100A7-9, which showed progressively greater expression in chronic lesions of AD[31]. These abovementioned genes deserve further investigation since they are potentially involved in the chronicity of AD.

In addition, our study was conducted with mice, and we did not perform RNA-seq in either mice or humans. Comparison of unbiased genome-wide gene expression between humans and mice would greatly promote research on the acute-to-chronic progression of AD.

## Methods

### Drugs and chemicals

Ovalbumin was purchased from Sigma‒Aldrich (Saint Louis, Missouri, 63,103, USA). Dinitrofluorobenzene was purchased from Macklin (Shanghai, 201,203, China). Antibodies used for immunohistochemistry were purchased from Abcepta (San Diego, California, 92,121, USA). Other required chemicals were purchased from Beyotime (Shanghai, 201,611, China). The concentration of DNFB was 0.15%, and a 3:1 mixture of acetone and olive oil was used as the solvent. The 0.15% ovalbumin solution was prepared with normal saline. All the drugs and chemicals used in this study were of analytical grade.

### Animals

Six-week-old female BALB/c mice weighing 20–25 g were procured from the Experimental Animal Centre of the Army Medical University (Third Military Medical University, Chongqing, 400,038, China). All the animals were housed in polypropylene cages and were given unlimited access to sterilized fodder and water. All experimental procedures were approved by the Laboratory Animal Welfare and Ethics Committee of Army Medical University (AMUWEC20223374). Animals were acclimatized to feeding conditions for 1 week before experimentation. At specific time points, the mice were sacrificed by cervical dislocation.

### Experimental design

Mice were divided into four groups as listed below.

**Fig. 7:**
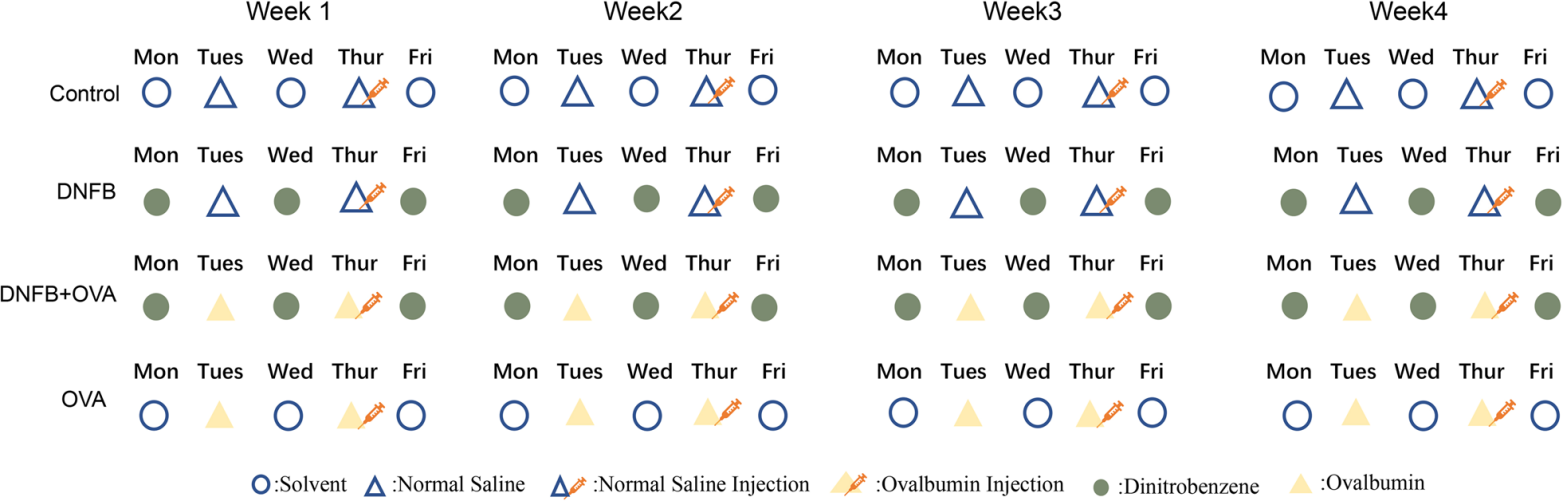
Sensitization and induction protocol. Mice were randomly divided into four groups, listed as below. Control group (n = 4): mice were given solvent or saline, no hapten, allergen or drugs were given to them. DNFB only group (n = 4): DNFB(100ul each time) and saline were given to them,5 times in total for each single week. DNFB + OVA group (n = 8): DNFB and OVA were alternately given to the mice 5 times in total for each week, four mice of this group were euthanized at day14 (acute phase) by cervical dislocation, the other four mice were euthanized by the same way at day28 (chronic phase). OVA only group (n = 4): OVA and solvent were alternately given to the mice 5 times in total each week

1. Control (NC) group (n = 4): Mice were given solvent or saline, and no hapten, allergen or drugs were given to them.
2. Dinitrofluorobenzene (DNFB)-only group (n = 4): Dinitrofluorobenzene (100 µl each time) and saline were given to the mice as detailed in Fig. 7.
3. Ovalbumin (OVA)-only group (n = 4): Ovalbumin (100 µl each time) and solvent were given to the mice as per the details given in Fig. 7.
4. Dinitrofluorobenzene + Ovalbumin (D + O) group (n = 8): DNFB and OVA were administered to the mice three times a week and two times a week, respectively. Both drugs were administered in 100 µl each time (see Fig. 7 for detailed information). Four of the mice were euthanized at Day 14 by cervical dislocation, and the other four mice were euthanized in the same way at Day 28.

### Sensitization protocol

After acclimation to the laboratory conditions, the back skin hair of all the mice was shaved with an electric razor, and the bare skin was treated with hair removal cream. The application of hair removal cream was repeated every three days to keep the skin clean. One hundred microliters of DNFB or solvent was uniformly smeared onto the shaved back of the mice three times a week. One hundred microliters of ovalbumin or saline was given twice a week, once for application to the shaved back skin, and once for intraperitoneal injection. Animals were sacrificed 24 h after the last exposure to reagents for procurement of blood samples and skin tissue. All reagents were softly and evenly applied to the mice. Cages were sanitized and replenished with chow and water weekly.

### Skin lesion evaluation

There are five main items to be scored: erythema, scaling, dryness/scarring, edema, and exudation. Each item has four grades, namely, 0 (none), 1 (mild), 2 (moderate), or 3 (severe). The total dermatitis score was calculated as the sum of these individual scores. The score of each item was assessed by two independent observers. Skin lesion evaluation was performed every four days, and eight evaluations were performed at the end of the experiment.

### Scratching behavior estimation

In addition to skin lesion evaluation, the scratching behavior estimation was also conducted at the end point of the experiment. The scoring criteria for scratching referred to a previous study [11]. Briefly, estimation was performed at one-minute intervals by an observer who was unaware of the treatment status. Estimation was performed for five consecutive minutes, and the scoring criteria were as follows: 1 if the scratching time duration was less than 1 s, 2 if the duration was less than 2 s, and 3 if the duration was more than 2 s. The scratching behavior estimation was performed 24 h after the last exposure to reagents.

### Histological examination

Skin specimens were fixed in 10% neutral formalin and embedded in paraffin. Slides of 5-µm-thick sections were prepared and stained with H&E to examine the epidermal thickness and inflammatory cell infiltration. Toluidine blue staining was performed to examine the infiltration of mast cells. Slides were then digitally scanned and analyzed using Iviewer Analysis Software (UNIC Technologies, Beijing, China). Mast cell counts and epidermal/dermal thickness measurements were performed in five randomly selected fields from each sample.

### Trans-epidermal water loss assay

Trans-epidermal water loss (TEWL) measurements were conducted with a Cutometer Dual MPA580 (Courage + Khazaka electronic Gmbh, Köln, Germany) to examine skin barrier dysfunction. Each mouse was measured 10 times in a randomly selected area.

### RNA isolation and quantitative real-time PCR

Total RNA was isolated from skin tissues using TRIzol™ reagent according to the manufacturer’s instructions. cDNA synthesis was performed using the PrimeScript™ RT Reagent Kit (TaKaRa, Japan) according to the manufacturer’s instructions. The expression values of each target gene were normalized to the expression values of β-actin. Real-time PCR was performed on a CFX Connect™ Real-Time System (BIO-RAD, CA, USA) with TB Green Premix Ex Taq™ II (TaKaRa, Japan). The amplification programs involved 40 cycles of preincubation for 2 min at 95 °C and then amplification cycles consisting of 5 s at 95 °C and 30 s at 60 °C. The analyzed genes and primer sequences of these genes are given in Table 1.

**Table 1:** Primer sequences

Gene	NCBI ID	Forward primer (5’-3’)	Reverese primer (5’-3’)
IL2	NM_0083663	TCACCCTTGCTAATCACTCCTC	TCCTGTAATTCTCCATCCTGCT
IL4	NM_021283.2	GATAAGCTGCACCATGAATGAGT	CCATTTGCATGATGCTCTTTAGG
IL13	NM_008355.3	TGGCTCTTGCTTGCCTTGGT	ACTCCATACCATGCTGCCGTT
IL19	NM_001009940.2	AGAGCCATGCAAACTAAGGACAC	GATCCTAGTTGCATTGGTGGCTT
IL22	NM_016971.2	CTTGTGCGATCTCTGATGGCT	CCAGCATAAAGGTGCGGTTG
IL4RA	NM_001008700.4	GCATCCCGTTGTTTTGCCTG	TTGGTTGACTCCTGGCTTCG
IL13RA1	NM_133990.5	ACAAGCCCTGACACACACTATAC	TCCCAGCATTATCCTTGACCATTAT
IL17A	NM_010552.3	CACCGCAATGAAGACCCTGATA	CAGCATCTTCTCGACCCTGAAAG
JAKI	NM_146145.2	CGGAACTTCCCAAAGACATCA	TCCAAGGTAGCCAGGTATTTCA
STAT3	NM_011486.5	TTTAACATTCTGGGCACGAACAC	ACGATCAAGGAGGCATCACAATT
STAT6	NM_009284.2	TCGGAAGCAGGAAGAACTCAAG	TTGGACCAGGACCATTGACAG
IFNγ	NM_008337.4	ATGAACGCTACACACTGCATCTT	TGACTGTGCCGTGGCAGTAA
IGFL1	NM_001111274.1	TGTCGTCTTCACACCTCTTCTAC	ACATCTCCAGTCTCCTCAGATCA
LORICRIN	NM_008508.3	GTGCTTCAGGGTAACCCTTCTC	AGAGGTCTTTCCACAACCCACA
FILAGGRIN	XM_017319842.1	CTGGGAGGCAAGCTACAACAACT	GTCTGCTCTGGGTCTTCTGTTTC
TSLP	NM_021367.2	CGACAAAACATTTGCCCGA	GCCATTTCCTGAGTACCGTCA
β-actin	NM_007393.5	GCATCCCGTTGTTTTGCCTG	TTGGTTGACTCCTGGCTTCG

### Enzyme-linked immunosorbent assay

The next day after the final exposure to reagents, blood was taken from the eyeballs of mice, and the serum was separated. Frozen serum samples were used to measure the serum levels of IL-2, IL-4, IL-13, IFN-γ, TARC, total IgE and OVA-specific IgE. ELISA was performed with commercial ELISA kits (JM-02339M1, JM-03061M1, JM-02981M1, JM-02448M1, JM-02456M1, and J2291-A; Jingmei Biology Technology, Jiangsu, China) according to the manufacturer’s instructions.

### Immunohistochemistry

Immunohistochemical staining was performed for filaggrin. The primary and secondary antibodies used were as follows: filaggrin (Host: rabbit; Reactivity: human, mouse, rat; Abcepta, San Diego, CA, 92,121, USA), and HRP-conjugated goat anti-rabbit IgG (H&L) (Source: goat; Immunogen: rabbit IgG; Conjugate: HRP; Abcepta, San Diego, CA, 92,121, USA). Tissues were fixed in 10% formalin at 4 °C overnight and were then embedded in paraffin. Next, 5-µm-thick sections were cut and routinely dewaxed and hydrated. Endogenous peroxidase activity and endogenous avidin binding activity were blocked. Sections were incubated with primary antibodies for 12 h at 4 °C overnight, and then the slides were rinsed three times in PBS for five minutes each wash. Sections were incubated with secondary antibodies for 30 min at 37 °C. Slides were counterstained with hematoxylin. Positively stained cells were counted from five random fields (magnification ⋅400) in each section.

### Statistical analysis

Data are expressed as the mean ± standard error of the mean or standard deviation. Statistical analysis was performed by Student’s t test or one-way analysis of variance (ANOVA) using GraphPad Prism software (GraphPad Software, Inc. La Jolla, CA, USA). Statistical significance was defined as *P*<0.05. The significance levels of the data are denoted as *P < 0.05, **P < 0.01, ***P < 0.001, and ****P<0.0001.

## Data Availability

The data and materials that support the findings of this study are available from the corresponding author upon reasonable request.

## Abbreviations

AD: Atopic Dermatitis
DNFB: Dinitrofluorobenzene
OVA: Ovalbumin
TEWL: Trans-epidermal water loss

## References

[1] Odhiambo JA, Williams HC, Clayton TO, Robertson CF, Asher MI. ISAAC Phase Three Study Group. Global variations in prevalence of eczema symptoms in children from ISAAC Phase Three. J Allergy Clin Immunol. 2009;124(6):1251-8.e23. doi: 10.1016/j.jaci.2009.10.009. PMID: 20004783. 10.1016/j.jaci.2009.10.009 20004783

[2] Silverberg JI, Hanifin JM (2013). Adult eczema prevalence and associations with asthma and other health and demographic factors: a US population-based study. JAllergy Clin Immunol..

[3] Kapoor R, Menon C, Hoffstad O, Bilker W, Leclerc P, Margolis DJ (2008). The prevalence of atopic triad in children with physician-confirmed atopic dermatitis. J Am Acad Dermatol..

[4] Brunner PM, Guttman-Yassky E, Leung DY (2017). The immunology of atopic dermatitis and its reversibility with broad-spectrum and targeted therapies. J Allergy Clin Immunol..

[5] Guttman-Yassky E, Nograles KE, Krueger JG (2011). Contrasting pathogenesis of atopic dermatitis and psoriasis–part I: clinical and pathologic concepts. J Allergy Clin Immunol.

[6] Chen L, Martinez O, Overbergh L, Mathieu C, Prabhakar BS, Chan LS (2004). Early up-regulation of Th2 cytokines and late surge of Th1 cytokines in an atopic dermatitis model. Clin Exp Immunol.

[7] Gandhi NA, Bennett BL, Graham NM, Pirozzi G, Stahl N, Yancopoulos GD (2016). Targeting key proximal drivers of type 2 inflammation in disease. Nat Rev Drug Discov.

[8] Nakajima S, Nomura T, Common J, Kabashima K (2019). Insights into atopic dermatitis gained from genetically defined mouse models. J Allergy Clin Immunol..

[9] Spergel JM, Mizoguchi E, Brewer JP, Martin TR, Bhan AK, Geha RS (1998). Epicutaneous sensitization with protein antigen induces localized allergic dermatitis and hyperresponsiveness to methacholine after single exposure to aerosolized antigen in mice. J Clin Invest..

[10] Szalai K, Kopp T, Lukschal A, Stremnitzer C, Wallmann J, Starkl P, Vander Elst L, Saint-Remy JM, Pali-Schöll I, Jensen-Jarolim E (2012). Establishing an allergic eczema model employing recombinant house dust mite allergens Der p1 and Der p 2 in BALB/c mice. Exp Dermatol.

[11] Porter RM (2003). Mouse models for human hair loss disorders. J Anat.

[12] Thomas WR (2012). House dust allergy and immunotherapy. Hum Vaccin Immunother.

[13] Brenninkmeijer EE, Schram ME, Leeflang MM, Bos JD, Spuls PI (2008). Diagnostic criteria for atopic dermatitis: a systematic review. Br J Dermatol.

[14] Bonneville M, Chavagnac C, Vocanson M, Rozieres A, Benetiere J, Pernet I, Denis A, Nicolas JF, Hennino A (2007). Skin contact irritation conditions the development and severity of allergic contact dermatitis. J Invest Dermatol..

[15] Kehren J, Desvignes C, Krasteva M, Ducluzeau MT, Assossou O, Horand F, Hahne M, Kägi D, Kaiserlian D, Nicolas JF (1999). Cytotoxicity is mandatory for CD8(+) T cell-mediated contact hypersensitivity. J Exp Med..

[16] Noda S, Suárez-Fariñas M, Ungar B, Kim SJ, de Guzman Strong C, Xu H, Peng X, Estrada YD, Nakajima S, Honda T, Shin JU, Lee H, Krueger JG, Lee KH, Kabashima K, Guttman-Yassky E (2015). The Asian atopic dermatitis phenotype combines features of atopic dermatitis and psoriasis with increased TH17 polarization. J Allergy Clin Immunol..

[17] Hagstrom EL, Patel S, Karimkhani C, Boyers LN, Williams HC, Hay RJ, Weinstock MA, Armstrong AW, Dunnick CA, Margolis DJ, Dellavalle RP (2015). Comparing cutaneous research funded by the US National Institutes of Health (NIH) with the US skin disease burden. J Am Acad Dermatol..

[18] David Boothe W, Tarbox JA, Tarbox MB (2017). Atopic dermatitis: pathophysiology. Adv Exp Med Biol..

[19] Feld M, Garcia R, Buddenkotte J, Katayama S, Lewis K, Muirhead G, Hevezi P, Plesser K, Schrumpf H, Krjutskov K, Sergeeva O, Müller HW, Tsoka S, Kere J, Dillon SR, Steinhoff M, Homey B (2016). The pruritus- and TH2-associated cytokine IL-31 promotes growth of sensory nerves. J Allergy Clin Immunol.

[20] Langan SM, Irvine AD, Weidinger S (2020). Atopic dermatitis. Lancet..

[21] Kashiwagi M, Hosoi J, Lai JF, Brissette J, Ziegler SF, Morgan BA, Georgopoulos K (2017). Direct control of regulatory T cells by keratinocytes. Nat Immunol..

[22] Adhikary PP, Tan Z, Page BDG, Hedtrich S (2021). TSLP as druggable target - a silver-lining for atopic diseases?. Pharmacol Ther..

[23] Klaeschen AS, Nümm TJ, Herrmann N, Leib N, Maintz L, Sakai T, Wenzel J, Bieber T (2021). JAK1/2 inhibition impairs the development and function of inflammatory dendritic epidermal cells in atopic dermatitis. J Allergy Clin Immunol.

[24] Kubo S, Nakayamada S, Sakata K, Kitanaga Y, Ma X, Lee S, Ishii A, Yamagata K, Nakano K, Tanaka Y (2018). Janus Kinase Inhibitor Baricitinib Modulates Human Innate and Adaptive Immune System. Front Immunol..

[25] Szalus K, Trzeciak M, Nowicki RJ (2020). JAK-STAT inhibitors in atopic dermatitis from pathogenesis to clinical trials results. Microorganisms..

[26] Bao L, Zhang H, Chan LS (2013). The involvement of the JAK-STAT signaling pathway in chronic inflammatory skin disease atopic dermatitis. JAKSTAT..

[27] He H, Guttman-Yassky E. JAK Inhibitors for Atopic Dermatitis: An Update. Am J Clin Dermatol. 2019;20(2):181–192. doi: 10.1007/s40257-018-0413-2. Erratum in: Am J Clin Dermatol. 2019 Jan 10;: PMID: 30536048. 10.1007/s40257-018-0413-2 30536048

[28] Tohyama M, Hanakawa Y, Shirakata Y, Dai X, Yang L, Hirakawa S, Tokumaru S, Okazaki H, Sayama K, Hashimoto K. IL-17 and IL-22 mediate IL-20 subfamily cytokine production in cultured keratinocytes via increased IL-22 receptor expression. Eur J Immunol. 2009 Oct;39(10):2779-88. doi: 10.1002/eji.200939473. PMID: 19731362. 10.1002/eji.200939473 19731362

[29] Lobito AA, Ramani SR, Tom I, Bazan JF, Luis E, Fairbrother WJ, Ouyang W, Gonzalez LC. Murine insulin growth factor-like (IGFL) and human IGFL1 proteins are induced in inflammatory skin conditions and bind to a novel tumor necrosis factor receptor family member, IGFLR1. J Biol Chem. 2011 May 27;286(21):18969–81. doi: 10.1074/jbc.M111.224626. Epub 2011 Mar 31. PMID: 21454693; PMCID: PMC3099712. 10.1074/jbc.M111.224626 PMC3099712 21454693

[30] Tsoi LC, Rodriguez E, Stölzl D, Wehkamp U, Sun J, Gerdes S, Sarkar MK, Hübenthal M, Zeng C, Uppala R, Xing X, Thielking F, Billi AC, Swindell WR, Shefler A, Chen J, Patrick MT, Harms PW, Kahlenberg JM, Perez White BE, Maverakis E, Gudjonsson JE, Weidinger S (2020). Progression of acute-to-chronic atopic dermatitis is associated with quantitative rather than qualitative changes in cytokine responses. J Allergy Clin Immunol.

[31] Gittler JK, Shemer A, Suárez-Fariñas M, Fuentes-Duculan J, Gulewicz KJ, Wang CQ, Mitsui H, Cardinale I, de Guzman Strong C, Krueger JG, Guttman-Yassky E (2012). Progressive activation of T(H)2/T(H)22 cytokines and selective epidermal proteins characterizes acute and chronic atopic dermatitis. J Allergy Clin Immunol.

